# Co-Design and Co-Delivery: The Benefits of Integration From the Family Caregiver’s Perspective

**DOI:** 10.5334/ijic.4225

**Published:** 2018-10-15

**Authors:** Jodeme Goldhar

**Affiliations:** 1 Executive Lead, Strategy and Innovation, The Change Foundation, Ontario, CA

**Keywords:** Integrated care, co-design, co-production, patients, clients, caregivers

Taking the service user’s perspective is the guiding principle of integrated care, but putting it into practice still poses many challenges. It doesn’t suffice to see the services through the patients’ eyes. To develop an integrated care plan FOR the patient and caregiver, without inclusion of their ideas and those of their family does not constitute participatory care. What we need is a shift from “doing to” to “doing with”, from “thinking for them” to “designing together”. Crucially, this involves a change of language, and an end to talking about “we”, the experts, professionals, integrated care proponents, versus “them”, the patient and caregiver, citizen, family, community. While nobody contests the importance of patient and caregiver involvement, acting upon it in reality is often impeded by learned behaviours, adverse organizational cultures or lack of training. However, as long as this radical shift in understanding does not occur, Carol Ann’s story below will keep repeating itself. This editorial illustrates the challenge to all of us, to reimagine health care and its systems through the eyes of patients and their caregivers because the systems were not designed with this in mind, and service providers, because no one ever thought of asking the family involved. It calls for a radical shift in understanding citizen involvement, and offers a way forward to finally deliver on the guiding principle of integrated care: that the person, family and community are at the centre of care.

As health systems around the world wrestle with the question of integration, the answer lies with patients and their families.

Patients and caregivers know the most about the physical, emotional, and spiritual aspects of their lives. Through their experiences, they also come to be well versed on when the healthcare system, including policy, funding, organization and practice decisions, do right by them, and also when it fails them.

Patients and caregivers can offer solutions and lead us to the improvements we truly need to make to our health care system. Yet, too often, solutions are not co-designed, and patient and caregiver experiences and expertise are not sought out.

The Change Foundation was asked to give a keynote address at the International Foundation for Integrated Care (IFIC) annual conference in Utrecht, Netherlands in May 2018.

Within my role as IFIC Senior Associate, and the focus to bring partnerships with patients and families into discussions on driving integrated systems of care, I wanted to truly convey a message of co-design, and person and family-centered care.

We, at The Change Foundation, knew this could not be done without the voice of caregivers, and we realized that we couldn’t authentically give a keynote unless we partnered with a patient/caregiver to co-design and co-deliver the keynote address.

So, together with caregiver Carole Anne Alloway, we undertook this process. We titled our presentation: *Transformation Through Integration: The Answer is With Patients and Their Families.*

## Our message

Underpinning the message we wanted to convey was our intent to shine a spotlight on the lived experience of care for patients and their caregivers. We wanted the audience to walk a step or two through Carole Ann Alloway experience. We wanted the audience to experience the frustration of a patient and caregiver on their journey. **However,** we also wanted to give examples of others who are **breaking new ground by improving their health systems** by engaging patients and their families in driving integration and illustrates how to so design with patients and their caregivers regardless of the lens you work from: policy, practice and research.

At The Change Foundation, we experienced this breakthrough when Carole Ann told her journey through the health care system. She listed what she had expected to have happen—things that in retrospect seem obvious and simple, but were not done.

This list of small things would have made a world of difference to Carole Ann, and The Change Foundation later based our caregiver wishlist project on it.

I spoke with Carole Ann after the presentation, to share our thoughts.

**Jodeme:** Carole Ann, what was your reaction when you were asked to co-present at the IFIC conference?

**Carole Ann:** It was overwhelming at first because there are so many things I wanted to say, but to get the most value for the audience, I realized we had to choose a key focus. With The Change Foundation’s knowledge, background and guidance, we started brainstorming the key messages and the approach to convey them to the audience in the best way.

What was your perspective on how to approach preparing the keynote, Jodeme?

**Jodeme:** Many health systems around the world see health system integration as an opportunity for transformation and improvement, but the perspectives on how to go about this transformation vary. We believe that patients and caregivers need to be partners every step of the way, regardless of the lens. Our goal for this presentation was to be provocative and inspire people to know that patients and caregivers are the experts on the physical, emotional and spiritual aspects of their lives, and it is important for healthcare professionals, researchers, and policy makers to recognize the value and to develop relationships with patients and families.

With my expertise on integration and global views on system change–in addition having an understanding of the potential audience and how best to communicate the key messages—we created a story line. Within this we thought about how best to build and think about our presentation from a system, research, policy and practice lens. Together, we drafted the presentation until we both felt satisfied with the result.

How did you feel about the process and the product, Carole Ann?

**Carole Ann:** I felt heard and valued for my lived experience. With your help my message got across in a way the audience understood. I didn’t want to just tell my story, I wanted people to leave with a sense of purpose to reflect on their own work habits, cultures and see how they can respond to what was possible by including their own patients and caregivers in the decision-making process and as a member of the team.

Jodeme, what do you feel the audience heard?

**Jodeme:** You didn’t just tell your story, you also re-told your story as if your husband’s care had been integrated and the team was focused on what was most important to you and your husband—the problems that could have been avoided, health care dollars saved, outcomes that could have been improved, as well as the experience for you, your husband, and the health care providers themselves.

You wanted the audience to leave knowing that this isn’t hard to do, the answer is simple and everyone can make a difference tomorrow regardless of where they work in their health care systems. Partnering with patients and their families is important if you are a researcher, funder, policy maker, healthcare organization and healthcare provider. And I truly think the audience heard that.

For us, it was important to be clear on what co-design is not so we can be clear on what it is. Often the clarity is not named and then people believe they are meaningfully co d-designing when in fact, people are sharing information as an example.

- It is not having a patient or a caregiver tell their story without it leading to change
- It is not bringing solutions to patients and families for their feedback
- It is not setting up a patient and family advisory committee for the sake of having it
- It is not professionalizing patients and families

**Figure:**
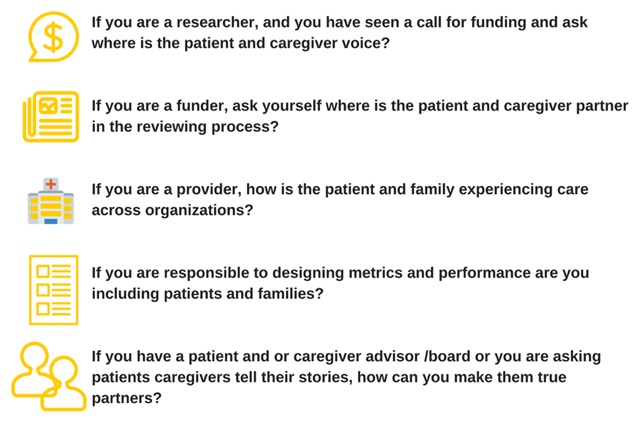


We are sometimes scared to meaningfully engage patients and their families in co-designing care, research, policy and practice together. We’re scared because we don’t know how, or that we won’t be able to meet the expectations of patients and families. We’re scared because in our busy practices, we’re not sure how to find the time to engage in a meaningful way. We have learned that it is not necessarily hard, but that it takes time and requires a shift in our mindsets and how we make change happen. It’s hard because our priorities can sometimes clash with the priorities of patients and their families. The thing is, though, that when we listen to patients and their families, it can serve as a guide for us. It can point us to the right answers for care, the right frame for our research and the right policies to enable the change that is required. We don’t have all the answers. Let’s start there. And then let’s turn towards patients and their families.

